# Postcardiac Arrest Care Delivery in Pediatric Intensive Care Units: A Plan and Call to Action

**DOI:** 10.1097/pq9.0000000000000727

**Published:** 2024-05-09

**Authors:** Jessica A. Barreto, Jesse Wenger, Maya Dewan, Alexis Topjian, Joan Roberts

**Affiliations:** From the * Department of Cardiology, Division of Cardiovascular Critical Care, Boston Children’s Hospital, Boston, Ma.; † Department of Pediatrics, Division of Critical Care Medicine, Seattle Children’s Hospital, Seattle, Wash.; ‡ Department of Pediatrics, Division of Critical Care Medicine, Cincinnati Children’s Hospital Medical Center, Cincinnati, Ohio; § Department of Anesthesia and Critical Care Medicine, Division of Critical Care Medicine, The Children’s Hospital of Philadelphia, Philadelphia, Pa.

## Abstract

**Background:**

Despite national pediatric postcardiac arrest care (PCAC) guidelines to improve neurological outcomes and survival, there are limited studies describing PCAC delivery in pediatric institutions. This study aimed to describe PCAC delivery in centers belonging to a resuscitation quality collaborative.

**Methods:**

An institutional review board-approved REDCap survey was distributed electronically to the lead resuscitation investigator at each institution in the international Pediatric Resuscitation Quality Improvement Collaborative. Data were summarized using descriptive statistics. A chi-square test was used to compare categorical data.

**Results:**

Twenty-four of 47 centers (51%) completed the survey. Most respondents (58%) belonged to large centers (>1,000 annual pediatric intensive care unit admissions). Sixty-seven percent of centers reported no specific process to initiate PCAC with the other third employing order sets, paper forms, or institutional guidelines. Common PCAC targets included temperature (96%), age-based blood pressure (88%), and glucose (75%). Most PCAC included electroencephalogram (75%), but neuroimaging was only included at 46% of centers. Duration of PCAC was either tailored to clinical improvement and neurological examination (54%) or time-based (45%). Only 25% of centers reported having a mechanism for evaluating PCAC adherence. Common barriers to effective PCAC implementation included lack of time and limited training opportunities.

**Conclusions:**

There is wide variation in PCAC delivery among surveyed pediatric institutions despite national guidelines to standardize and implement PCAC.

## INTRODUCTION

Following resuscitation from cardiac arrest, patients experience postcardiac arrest syndrome defined by brain injury, myocardial dysfunction, and systemic reperfusion injury.^1^ Postcardiac arrest care (PCAC) is a guideline-based approach that includes targeted temperature management, monitoring for and treating seizures, targeting normal oxygenation and ventilation, preventing hypotension, and maintaining normoglycemia.^1^ There are conflicting data on the survival effect of individual PCAC elements, and no studies have evaluated their joint effect as a bundle of care.^1^ Clinical trials on targeted temperature management found no difference in survival with a good functional outcome between therapeutic hypothermia and therapeutic normothermia.^2,3^ Despite the high frequency of seizures after cardiac arrest,^4,5^ there is insufficient evidence linking the treatment of seizures with outcomes.^1^ Although hypoxemia is generally thought to be harmful, there have been inconsistent differences in survival with hypoxemia, hyperoxemia, hypocapnia, and hypercapnia.^6–8^ Hypotension is associated with lower survival after cardiac arrest,^9,10^ but the survival effect of manipulating blood pressure after arrest remains unclear.^1^ Finally, although hypoglycemia and hyperglycemia are associated with unfavorable outcomes in critically ill children,^11^ no clinical trials have focused on the postarrest period.^1^

The 2020 American Heart Association (AHA) Pediatric Advanced Life Support guidelines^12^ and the scientific statement on pediatric PCAC^1^ provide goals and management strategies for postarrest care based on consensus among pediatric and adult specialists and a review of the past 20 years of literature. It is unknown how these national guidelines have been implemented into local PCAC practice.^12^ A survey of emergency hospitals treating adults after cardiac arrest reported that less than half of centers had local guidelines for PCAC. Of those institutions with local guidelines, 39% reported that the guidelines were not always applied.^13^ Similar studies have not been published on children, and there is a lack of data on providers’ perceptions of barriers to implementation. The purpose of this study is to describe the reported variation of PCAC delivery in intensive care units (ICUs) participating in the Pediatric Resuscitation Quality (PediRES-Q) Collaborative (ClinicalTrials.gov NCT02708134).

## METHODS

The PediRES-Q collaborative comprises pediatric ICUs from institutions worldwide that vary in size, geographic location, and academic support and share a collective purpose of conducting research and quality improvement (QI) projects to improve pediatric resuscitation.^14^ Surveys were distributed electronically using REDCap to resuscitation investigators at institutions belonging to PediRES-Q (Richmond, Va.). REDCap is a secure web application for managing surveys and databases.^15^ The investigators responding to the surveys were pediatric intensivists from Pediatric Intensive Care Units (PICUs) or mixed units (combined PICU and cardiac ICU) who regularly attend monthly PediRES-Q meetings and are directly involved with PCAC at their institution.

The survey included closed and open-ended questions organized into four sections: characteristics of the institution, PCAC implementation and understanding, PCAC bundle components, and PCAC adherence. Survey questions were assessed for readability, clarity, and inclusive response options on a group of volunteer investigators belonging to PediRES-Q before distributing electronically to the primary investigator at each of the 47 centers. The collaborative sent two reminder e-mails and announcements were made at two of the monthly videoconferences. Not all survey questions were mandatory, and we included partially completed surveys in the analysis. The institutional review board of the Children’s Hospital of Philadelphia deemed the study exempted. Data are summarized using descriptive statistics, and chi-square testing was used to compare groups. Statistical analysis was performed with R Statistical Software version 4.0.4 (Vienna, Austria).

## RESULTS

Twenty-four of 47 (51%) centers completed the survey. More than half of centers (14/24, 58%) have more than 1,000 annual PICU admissions, 11 of 24 (46%) centers have more than 30 PICU beds and 16 of 24 (67%) centers have extracorporeal cardiopulmonary resuscitation available at their institution. Most institutions have trainees (23/24, 96%) and advanced practice providers (19/24, 79%) involved in delivering care. The reported primary method of PCAC initiation at most centers (16/24, 67%) was by attending physician orders without the assistance of order sets, paper forms, or institutional guidelines. This method was more common in smaller (<1,000 admissions) versus larger (>1000 admissions) PICUs (80% versus 57%, *P* = 0.04). Sixteen (67%) centers reported not using specific selection criteria to initiate PCAC, such as duration of cardiopulmonary resuscitation or neurological examination (Table 1).

**Table 1.** Characteristics of PCAC initiation

Characteristic (n = 24)	n (%)
Location	
Type of ICU where PCAC is initiated	
PICU	17 (71)
CICU/CVICU	5 (21)
Mixed unit (PICU and CICU)	2 (8)
NICU	0 (0)
PCAC initiated before arrival to the ICU	6 (25)
Location where PCAC can be initiated*	
Emergency room	4 (17)
Transport	4 (17)
Operating room	2 (8)
Hospital ward	2 (8)
Process	
Tools used for PCAC initiation in the ICU	
No (PCAC initiated by attending orders without other tools)	16 (67)
Yes*	8 (33)
Order set	8 (33)
Paper form	7 (29)
Team huddle	9 (38)
Institutional policy/guideline of care	9 (38)
Dedicated postarrest team	2 (8)
None	9 (38)
Goal timing of initiation after admission to the ICU	
0–1 h	11 (46)
1–2 h	4 (17)
No goal	9 (38)
Selection criteria	
Specific selection criteria used to initiate PCAC	
No	16 (67)
Yes	8 (33)
CPR duration >60 s before ROSC	3 (13)
GCS or other neurological scale threshold†	5 (21)

* Respondents were able to select more than one option.

† Three of five respondents reported using specific GCS levels: GCS 2 (one respondent), GCS 6 (two respondents).

CICU, cardiac intensive care unit; GCS, Glasgow coma scale; NICU, neonatal intensive care unit.

Most centers reported having established temperature (23, 96%), glucose (18, 75%), and age-based blood pressure (21, 88%) PCAC goals. Only 9 (38%) centers report intentional regulation of sodium in the normal to hypernatremia range (>135), of which eight use hypertonic saline to achieve sodium goals. Neurological monitoring with electroencephalogram was reported by 19 (75%) centers. Brain imaging in the first 72 hours after arrest was reported in 11 (46%) centers, with computed tomography and magnetic resonance imaging being the most common modalities (6, 25% and 7, 29%) and transcranial Doppler being the least common (2, 8%). Of note, three (13%) centers reported not using neurological monitoring or imaging during the first 72 hours after arrest. PCAC duration is highly variable, with most centers tailoring duration to clinical improvement and neurological exam (13, 54%), followed by 72 hours (7, 29%), 48 hours (2, 8%), and 24 hours (2, 8%) (Table 2).

**Table 2.** PCAC components

Characteristic (n = 24)	Yes, n (%)
PCAC duration, h	
24	2 (8)
48	2 (8)
72	7 (29)
96	2 (8)
Variable duration*	13 (54)
Temperature goals	
Avoidance of fever (<38 °C)	7 (29)
Controlled normothermia (36 °C–37.5 °C) for 5 d	7 (29)
Induced hypothermia (32 °C–34 °C for 48 h), then controlled normothermia for 72 h	0 (0)
Mixture of both controlled normothermia and induced hypothermia	8 (33)
Use of different temperature goals for IHCA vs OHCA†	1 (4)
None	1 (4)
Metabolic goals	
Intentional regulation of glucose	18 (75)
Lower limit targeted, mg/dL	
60	3 (13)
70	4 (17)
80	3 (13)
100	6 (25)
Other	2 (8)
Upper limit targeted, mg/dL	
180	4 (17)
200	11 (46)
Other	3 (13)
Intentional regulation of sodium	9 (38)
Lower limit targeted, mg/dL	
135	3 (13)
140	5 (21)
145	1 (4)
Use of hypertonic saline to achieve sodium goals	8 (33)
Hemodynamic goals	
Use of age-based BP goals	21 (88)
MAP goal established	19 (79)
>5th percentile	4 (17)
>50th percentile	4 (17)
Attending discretion (no institutional standard)	9 (38)
Other‡	2 (8)
SBP goal established	20 (83)
>5th percentile	5 (21)
>10th percentile	1 (4)
>50th percentile	3 (13)
Attending discretion (no institutional standard)	9 (38)
Other‡	2 (8)
Use of NIRS monitoring	12 (50)
Cerebral	12 (50)
Somatic/renal	9 (38)
Routine placement of arterial lines during PCAC§	20 (83)
For BP monitoring regardless of the need for pressors	13 (54)
For BP monitoring only if pressors required	7 (29)
Arterial blood gas sampling	14 (58)
Nurses able to titrate vasoactive medication to a BP goal without a provider changing the order	18 (75)
Neurological	
Use of EEG monitoring	19 (75)
Continuous EEG	17 (71)
Spot EEG	1 (4)
Other	1 (4)
Timing of EEG initiation after ROSC, h	
0–4	8 (33)
4–8	7 (29)
>8	2 (8)
Minimum duration of EEG monitoring, h	
<1	1 (4)
1–4	1 (4)
>12	12 (50)
Other	3 (13)
Use of transcranial Doppler examinations	2 (8)
Use of brain imaging during the immediate postarrest period§‖	11 (46)
CT	6 (25)
MRI	7 (29)
Haste MRI	0 (0)
Cranial ultrasound	2 (8)
Other¶	1 (4)
Goal time to obtain brain imaging after ROSC, h	
12	1 (4)
24	1 (4)
48	1 (4)
72	8 (33)

* Based on clinical improvement/neurological examination.

† Respondent indicated use of both controlled normothermia and induced hypothermia for OCHA and controlled normothermia for IHCA.

‡ Percentile used varies by age group.

§ Respondents were able to select more than one option.

‖ Respondents were asked to assume that 72 hours after ROSC is the immediate postarrest period.

¶ Respondent obtains CT on all patients with OHCA to look for cause of arrest on admission, then MRI at days 3–5 for all patients who are not back at baseline.

CT, computed tomography; EEG, electroencephalogram; IHCA, in-hospital cardiac arrest; MAP, mean arterial blood pressure; MRI, magnetic resonance imaging; NIRS, near-infrared spectroscopy; OHCA, out-of-hospital cardiac arrest; ROSC, return of spontaneous circulation; SBP, systolic blood pressure.

Most centers (17, 71%) have a method in place for training staff on PCAC implementation, most commonly through staff meetings (15, 63%) targeting attending physicians (16, 67%), trainees (14, 58%), advanced practice providers (11, 47%), and bedside nurses (11, 46%), but rarely respiratory therapists (2, 8%) and pharmacists (1, 4%). Only six centers (25%) reported having a mechanism for evaluating adherence. When asked to select the top three barriers that affect the implementation of PCAC, most respondents chose lack of time/competing obligations (20, 83%), lack of interest/important stakeholders (14, 58%), and limited training opportunities (8, 33%). Reported facilitators to improve PCAC implementation included availability of resources (immediate, continuous electroencephalogram monitoring, temperature regulation systems) and staff awareness (improved recognition of the importance of temperature monitoring and control, use of PCAC report cards, review of the AHA PCAC statement in formal educational settings, use of the PediRES-Q PREP checklist, implementation of an institutional cardiac arrest registry, and discussions during bedside rounds).

## DISCUSSION

This is the first study to describe pediatric intensivists’ reported PCAC implementation at pediatric institutions. There was considerable variability in reported PCAC implementation, with most centers relying on attending-directed care to implement and guide PCAC without standardized order sets, paper forms, or institutional guidelines. Most centers reported not using specific criteria to initiate PCAC. Additionally, PCAC duration was highly variable, with its length most often determined by clinical improvement/neurological examination and less frequently by set time intervals. Few centers have practices incorporating all components of postarrest care as suggested by Pediatric Advanced Life Support guidelines.

These findings are not surprising because other established care guidelines are also poorly adopted into reliable practice patterns.^16^ These pediatric institutions likely represent some of the most motivated and best-resourced pediatric institutions (67% have extracorporeal cardiopulmonary resuscitation programs) in caring for pediatric cardiac arrest patients. Yet, these institutions still report high variability in how PCAC is delivered. Similar to infection prevention bundles to prevent line-associated infections (central line-associated bloodstream infection),^17^ we suggest PCAC may be best delivered as a bundle with the benefit derived from its multiple elements, and likely when parts of the bundle are missing, the efficacy of PCAC decreases.^18,19^ Yet, most institutions reported incomplete implementation of all components of PCAC. Finally, evaluation of bundle adherence, a key component of care bundle implementation and effectiveness,^20^ was notably low across centers.

### Next Steps

The information we have learned on reported PCAC practices across pediatric ICUs (*what is*) highlights a need to better understand postarrest care perception and application to outline benchmarks for PCAC adherence (*what should be*) and to chart a path to meet those benchmarks (*how we get there*).

### What Should Be?

The AHA statement on pediatric PCAC delineates management strategies that should be instituted after cardiac arrest and serves as the sole reference for the US pediatric centers providing PCAC.^1^ In translating these recommendations to the bedside, several knowledge gaps persist, including understanding the optimal targets and therapies that affect outcomes, outlining ways to track the impact of PCAC, determining the effect of using a bundle of care, identifying the necessary team members and institutional features to influence outcomes, and defining the best measures to define these outcomes.^1^

Thus far, PCAC clinical trials evaluating postarrest outcomes have focused exclusively on temperature management (eg, target temperature and duration).^2,21^ No other PCAC components, nor the bundle, have been evaluated in a clinical trial. Lack of clarity on the effectiveness of PCAC components and the PCAC bundle is a significant barrier to establishing postarrest care quality indicators, which have thus far not been delineated in the pediatric population. Such quality metrics were recently published for PCAC after adult out-of-hospital arrest.^22^ The absence of quality metrics and benchmarks hinder the implementation of QI initiatives and is a necessary step in improving PCAC delivery.

### How We Get There?

Successful implementation of QI initiatives must focus on understanding the intervention’s effects and the contextual factors that influence implementation.^23^ Thus, the first step in improving PCAC delivery is to leverage cardiac arrest databases to fill existing knowledge gaps. Available registries and collaboratives include PediRES-Q,^24^ AHA—Get With The Guidelines,^25^ Therapeutic Hypothermia After Cardiac Arrest,^2^ Pediatric Cardiac Critical Care Consortium,^26^ Extracorporeal Life Support Organization,^27^ Cardiac Arrest Registry to Enhance Survival, and Canadian Resuscitation Outcomes Consortium.^28^ Data sharing agreements, direct reporting from electronic medical records, and shared definitions for data elements of these databases would greatly enhance participation to defray the cost and resources required to participate.

The second step is to understand the facilitators and barriers to implementing QI initiatives, which as highlighted by Dewan et al,^23^ are not unique to postarrest care. In their study of contextual factors affecting the implementation of resuscitation QI interventions in centers belonging to pediRES-Q, the authors highlight disparities in center performance despite universal access to the same interventions. Facilitators of QI implementation included a unified institutional approach to QI, a focus on learning from failures and trying new approaches, leadership support beyond the divisional level, strong motivation to improve, knowledge sharing with key stakeholders at other institutions, and prioritization of goals. Barriers included low QI team tenure (eg, rotating medical providers), lack of resources and time, lack of formalized QI knowledge or training, and lack of buy-in from leaders and staff. Further understanding of contextual factors that influence QI success with the use of tools such as the Model for Understanding Success in Quality framework^29^ and application of this knowledge by strong local teams trained in implementation science and QI are key to improving PCAC delivery.^23^

The final step in improving PCAC delivery is to continue providing a unified approach for PCAC implementation and evaluation, such as that offered by pediRES-Q.^24^ The collaborative provides a publicly available postresuscitation care checklist outlining the management goals during the postarrest period that participating sites can print for use at the bedside.^30^ Furthermore, the collaborative provides a platform for data collection through which participating sites can submit data on PCAC delivery and compare center performance. The collaborative also serves as a place for knowledge sharing and support through monthly meetings and access to a network of providers at other institutions. Finally, the data and research infrastructure provided by the collaborative can be used for ongoing assessment of PCAC application through an implementation science approach while measuring its effectiveness on outcomes through a hybrid study design.

We encourage institutions to consider QI initiatives derived from locally identified PCAC barriers and facilitators. Based on the themes identified through our survey, some potential key drivers and interventions that could be implemented across multiple plan-do-study-act cycles include: improving institutional engagement (identification of PCAC champions at each institution with active participation in PediRES-Q meetings); improving staff understanding (periodic staff education and refreshers, structured review of cases where PCAC was used); increasing PCAC training opportunities (integration of PCAC care into cardiac arrest simulation); implementing PCAC tools (use of a bedside checklist such as the PediRES-Q PREP checklist,^30^ creation of an electronic medical record order set outlining PCAC interventions and goals, huddles during daytime and nighttime rounds to evaluate goals and compliance); and instituting mechanisms for evaluating PCAC adherence (use of PCAC report cards, data collection through institutional cardiac arrest registries, data submission to PediRES-Q). Statistical process control^31^ can be used to track process metrics (eg, PCAC adherence—defined as the percentage of bundle treatment goals achieved per cardiac arrest event) and outcome metrics (eg, survival and favorable neurological outcome) to inform future interventions.

### Limitations

There are several limitations to our study. This was a survey of physicians within an international collaborative with a modest response rate. Survey responses are what people say they do and may not reflect actual practice. These physicians and the institutions they represent may not portray the most common views because physicians interested in PCAC are likely overrepresented in this population. These physicians also have a potential for reporting bias given that the individuals responding to the surveys regularly participate in all stages of PCAC, and this may overrepresent how PCAC is delivered. Additionally, we did not perform systematic pretesting or validation of our survey before its administration, which may decrease the validity of our results.

## CONCLUSIONS

There is wide variation in PCAC delivery among the PICUs surveyed. We identified potential common themes and opportunities, such as standardizing PCAC initiation methods, bundle components, and training, adherence, and evaluation methods that QI and implementation science interventions could target to improve PCAC effectiveness. We believe that every pediatric institution that cares for children postcardiac arrest, should identify a PCAC champion who can lead the charge to help improve PCAC delivery.
